# The effect of social media on well-being differs from adolescent to adolescent

**DOI:** 10.1038/s41598-020-67727-7

**Published:** 2020-07-01

**Authors:** Ine Beyens, J. Loes Pouwels, Irene I. van Driel, Loes Keijsers, Patti M. Valkenburg

**Affiliations:** 1 Amsterdam School of Communication Research, University of Amsterdam, 1001 NG Amsterdam, The Netherlands; 2 Department of Developmental Psychology, Tilburg University, 5000 LE Tilburg, The Netherlands

**Keywords:** Psychology, Human behaviour

## Abstract

The question whether social media use benefits or undermines adolescents’ well-being is an important societal concern. Previous empirical studies have mostly established across-the-board effects among (sub)populations of adolescents. As a result, it is still an open question whether the effects are unique for each individual adolescent. We sampled adolescents’ experiences six times per day for one week to quantify differences in their susceptibility to the effects of social media on their momentary affective well-being. Rigorous analyses of 2,155 real-time assessments showed that the association between social media use and affective well-being differs strongly across adolescents: While 44% did not feel better or worse after passive social media use, 46% felt better, and 10% felt worse. Our results imply that person-specific effects can no longer be ignored in research, as well as in prevention and intervention programs.

## Introduction

Ever since the introduction of social media, such as Facebook and Instagram, researchers have been studying whether the use of such media may affect adolescents’ well-being. These studies have typically reported mixed findings, yielding either small negative, small positive, or no effects of the time spent using social media on different indicators of well-being, such as life satisfaction and depressive symptoms (for recent reviews, see for example^1–5^). Most of these studies have focused on between-person associations, examining whether adolescents who use social media more (or less) often than their peers experience lower (or higher) levels of well-being than these peers. While such between-person studies are valuable in their own right, several scholars^6, 7^ have recently called for studies that investigate within-person associations to understand whether an increase in an adolescent’s social media use is associated with an increase or decrease in that adolescent’s well-being. The current study aims to respond to this call by investigating associations between social media use and well-being within single adolescents across multiple points in time^8–10^.

### Person-specific effects

To our knowledge, four recent studies have investigated within-person associations of social media use with different indicators of adolescent well-being (i.e., life satisfaction, depression), again with mixed results^6, 11–13^. Orben and colleagues^6^ found a small negative reciprocal within-person association between the time spent using social media and life satisfaction. Likewise, Boers and colleagues^12^ found a small within-person association between social media use and increased depressive symptoms. Finally, Coyne and colleagues^11^ and Jensen and colleagues^13^ did not find any evidence for within-person associations between social media use and depression.

Earlier studies that investigated within-person associations of social media use with indicators of well-being have all only reported *average* effect sizes. However, it is possible, or even plausible, that these average within-person effects may have been small and nonsignificant because they result from sizeable heterogeneity in adolescents’ susceptibility to the effects of social media use on well-being (see^14, 15^). After all, an average within-person effect size can be considered an aggregate of numerous individual within-person effect sizes that range from highly positive to highly negative.

Some within-person studies have sought to understand adolescents’ differential susceptibility to the effects of social media by investigating differences between subgroups. For instance, they have investigated the moderating role of sex to compare the effects of social media on boys versus girls^6, 11^. However, such a group-differential approach, in which potential differences in susceptibility are conceptualized by group-level moderators (e.g., gender, age) does not provide insights into more fine-grained differences at the level of the single individual^16^. After all, while girls and boys each represent a homogenous group in terms of sex, they may each differ on a wide array of other factors.

As such, although worthwhile, the average within-person effects of social media on well-being obtained in previous studies may have been small or non-significant because they are diluted across a highly heterogeneous population (or sub-population) of adolescents^14, 15^. In line with the proposition of media effects theories that each adolescent may have a unique susceptibility to the effects of social media^17^, a viable explanation for the small and inconsistent findings in earlier studies may be that the effect of social media differs from adolescent to adolescent. The aim of the current study is to investigate this hypothesis and to obtain a better understanding of adolescents’ unique susceptibility to the effects of social media on their affective well-being.

### Social media and affective well-being

Within-person studies have provided important insights into the associations of social media use with cognitive well-being (e.g., life satisfaction^6^), which refers to adolescents’ cognitive judgment of how satisfied they are with their life^18^. However, the associations of social media use with adolescents’ affective well-being (i.e., adolescents’ affective evaluations of their moods and emotions^18^) are still unknown. In addition, while earlier within-person studies have focused on associations with trait-like conceptualizations of well-being^11–13^, that is, adolescents’ average well-being across specific time periods^18^, there is a lack of studies that focus on well-being as a momentary affective state. Therefore, we extend previous research by examining the association between adolescents’ social media use and their momentary affective well-being. Like earlier experience sampling (ESM) studies among adults^19, 20^, we measured adolescents’ momentary affective well-being with a single item. Adolescents’ momentary affective well-being was defined as their current feelings of happiness, a commonly used question to measure well-being^21, 22^, which has high convergent validity, as evidenced by the strong correlations with the presence of positive affect and absence of negative affect.

To assess adolescents’ momentary affective well-being (henceforth referred to as well-being), we conducted a week-long ESM study among 63 middle adolescents ages 14 and 15. Six times a day, adolescents were asked to complete a survey using their own mobile phone, covering 42 assessments per adolescent, assessing their affective well-being and social media use. In total, adolescents completed 2,155 assessments (83.2% average compliance).

We focused on middle adolescence, since this is the period in life characterized by most significant fluctuations in well-being^23, 24^. Also, in comparison to early and late adolescents, middle adolescents are more sensitive to reactions from peers and have a strong tendency to compare themselves with others on social media and beyond. Because middle adolescents typically use different social media platforms, in a complementary way^25–27^, each adolescent reported on his/her use of the three social media platforms that s/he used most frequently out of the five most popular social media platforms among adolescents: WhatsApp, followed by Instagram, Snapchat, YouTube, and, finally, the chat function of games^28^. In addition to investigating the association between overall social media use and well-being (i.e., the summed use of adolescents’ three most frequently used platforms), we examined the unique associations of the two most popular platforms, WhatsApp and Instagram^28^.

Like previous studies on social media use and well-being, we distinguished between active social media use (i.e., “activities that facilitate direct exchanges with others”^29^) and passive social media use (i.e., “consuming information without direct exchanges”^29^). Within-person studies among young adults have shown that passive but not active social media use predicts decreases in well-being^29^. Therefore, we examined the unique associations of adolescents’ overall active and passive social media use with their well-being, as well as active and passive use of Instagram and WhatsApp, specifically. We investigated categorical associations, that is, whether adolescents would feel better or worse if they had actively or passively used social media. And we investigated dose–response associations to understand whether adolescents’ well-being would change as a function of the time they had spent actively or passively using social media.

The hypotheses and the design, sampling and analysis plan were preregistered prior to data collection and are available on the Open Science Framework, along with the code used in the analyses (https://osf.io/nhks2). For details about the design of the study and analysis approach, see Methods.

## Results

In more than half of all assessments (68.17%), adolescents had used social media (i.e., one or more of their three favorite social media platforms), either in an active or passive way. Instagram (50.90%) and WhatsApp (53.52%) were used in half of all assessments. Passive use of social media (66.21% of all assessments) was more common than active use (50.86%), both on Instagram (48.48% vs. 20.79%) and WhatsApp (51.25% vs. 40.07%).

Strong positive between-person correlations were found between the duration of active and passive social media use (overall: *r* = 0.69, *p* < 0.001; Instagram: *r* = 0.38, *p* < 0.01; WhatsApp: *r* = 0.85, *p* < 0.001): Adolescents who had spent more time actively using social media than their peers, had also spent more time passively using social media than their peers. Likewise, strong positive within-person correlations were found between the duration of active and passive social media use (overall: *r* = 0.63, *p* < 0.001; Instagram: *r* = 0.37, *p* < 0.001; WhatsApp: *r* = 0.57, *p* < 0.001): The more time an adolescent had spent actively using social media at a certain moment, the more time s/he had also spent passively using social media at that moment.

Table 1 displays the average number of minutes that adolescents had spent using social media in the past hour at each assessment, and the zero-order between- and within-person correlations between the duration of social media use and well-being. At the between-person level, the duration of active and passive social media use was not associated with well-being: Adolescents who had spent more time actively or passively using social media than their peers did not report significantly higher or lower levels of well-being than their peers. At the within-person level, significant but weak positive correlations were found between the duration of active and passive overall social media use and well-being. This indicates that adolescents felt somewhat better at moments when they had spent more time actively or passively using social media (overall), compared to moments when they had spent less time actively or passively using social media. When looking at specific platforms, a positive correlation was only found for passive WhatsApp use, but not for active WhatsApp use, and not for active and passive Instagram use.

**Table 1:** Means, standard deviations, and zero-order between-person and within-person correlations for well-being and duration of use.

	*M* (*SD*)	Well-being between-person	Well-being within-person
Well-being	5.61 (0.75)	–	–
Duration of overall active social media use	12.47 (11.49)	.06	.09**
Duration of overall passive social media use	19.71 (8.95)	.17	.07*
Duration of active Instagram use	5.15 (6.69)	− .03	.04
Duration of passive Instagram use	9.39 (4.84)	− .07	.03
Duration of active WhatsApp use	5.34 (4.14)	.08	.04
Duration of passive WhatsApp use	7.34 (3.69)	.01	.09***

Means were calculated at the between-person level and represent the average number of minutes that adolescents had spent using social media in the past hour across assessments during which adolescents had used social media/Instagram/WhatsApp in the past hour*.* All correlations are based on the assessments during which participants had used social media/Instagram/WhatsApp, either actively or passively.* N* = 63 for active and passive overall social media use;* N* = 60 for active and passive Instagram use;* N* = 63 for active and passive WhatsApp use.

*p < .05. **p < .01. ***p < .001.

### Average and person-specific effects

The within-person associations of social media use with well-being and differences in these associations were tested in a series of multilevel models. We ran separate models for overall social media use (i.e., active use and passive use of adolescents’ three favorite social media platforms, see Table 2), Instagram use (see Table 3), and WhatsApp use (see Table 4). In a first step we examined the average categorical associations for each of these three social media uses using fixed effects models (Models 1A, 3A, and 5A) to investigate whether, on average, adolescents would feel better or worse at moments when they had used social media compared to moments when they had not (i.e., categorical predictors: active use versus no active use, and passive use versus no passive use). In a second step, we examined heterogeneity in the within-person categorical associations by adding random slopes to the fixed effects models (Models 1B, 3B, and 5B). Next, we examined the average dose–response associations using fixed effects models (Models 2A, 4A, and 6A), to investigate whether, on average, adolescents would feel better or worse when they had spent more time using social media (i.e., continuous predictors: duration of active use and duration of passive use). Finally, we examined heterogeneity in the within-person dose–response associations by adding random slopes to the fixed effects models (Models 2B, 4B, and 6B).

**Table 2:** Within-person associations between adolescents’ overall use of social media and well-being.

	Categorical associations Use versus no use (*N* participants = 63; *N* assessments = 2,155)							Dose–response associations Duration of use (*N* participants = 63; *N* assessments = 1,474)						
	Model 1A Fixed effects				Model 1B Random effects			Model 2A Fixed effects				Model 2B Random effects		
	*B*	(*SE*)	*p*	β	*B*	(*SE*)	*p*	*B*	(*SE*)	*p*	β	*B*	(*SE*)	*p*
**Fixed part**														
Intercept	5.60	(.10)	< .001	7.63	5.60	(.10)	< .001	5.66	(.09)	< .001	8.05	5.66	(.09)	< .001
Assessment (WP)	.09	(.04)	.020	.09	.09	(.04)	.015	.07	(.04)	.094	.07	.06	(.04)	.091
Passive use (WP)	.14	(.09)	.111	.06	.14	(.09)	.114	.03	(.03)	.270	.04	.03	(.03)	.243
Active use (WP)	.14	(.08)	.096	.05	.15	(.08)	.058	.07	(.04)	.040	.07	.07	(.04)	.053
**Random part**														
σ^2^ residual (WP)	1.16	(.11)	< .001		1.13	(.11)	< .001	1.10	(.11)	< .001		1.08	(.11)	< .001
σ^2^ between-person (BP)	.54	(.08)	< .001		.54	(.08)	< .001	.49	(.09)	< .001		.50	(.09)	< .001
σ^2^ passive use (BP)					.11	(.05)	.015					< .01	(.01)	.333
σ^2^ active use (BP)					.05	(.06)	.209					.01	(.01)	.221
**Model fit**														
Deviance	6,616.58				6,603.86			4,470.03				4,467.78		
AIC	6,628.58				6,619.86			4,482.03				4,483.78		
BIC	6,662.63				6,665.26			4,513.80				4,526.14		
Chi^2^ (df)					17.52 (2)		< .001					2.62 (2)		.270

For investigating the categorical associations, the passive and active use predictors were dummy coded (passive use: 0 = no passive use of social media; 1 = passive use of social media; and active use: 0 = no active use of social media; 1 = active use of social media, respectively). WP = within-person; BP = between-person. All predictors were person-mean centered. Models for the duration of use only include assessments during which participants had used social media, either actively or passively. *p*-values of the fixed part of the model are two-sided, *p*-values of the random part of the model are one-sided.

**Table 3:** Within-person associations between adolescents’ use of Instagram and well-being.

	Categorical associations Use versus no use (*N* participants = 60; *N* assessments = 2,112)							Dose–response associations Duration of use (*N* participants = 60; *N* assessments = 1,075)						
	Model 3A Fixed effects				Model 3B Random effects			Model 4A Fixed effects				Model 4B Random effects		
	*B*	(*SE*)	*p*	β	*B*	(*SE*)	*p*	*B*	(*SE*)	*p*	β	*B*	(*SE*)	*p*
**Fixed part**														
Intercept	5.64	(.10)	< .001	7.89	5.64	(.10)	< .001	5.69	(.10)	< .001	7.62	5.69	(.10)	< .001
Assessment (WP)	.08	(.04)	.031	.09	.08	(.04)	.033	.05	(.05)	.347	.09	.05	(.05)	.276
Passive use (WP)	.15	(.07)	.027	.14	.15	(.07)	.041	.03	(.07)	.695	.14	.06	(.07)	.440
Active use (WP)	.14	(.09)	.137	.13	.13	(.09)	.164	.05	(.06)	.428	.06	.01	(.07)	.879
**Random part**														
σ^2^ residual (WP)	1.18	(.11)	< .001		1.17	(.10)	< .001	1.11	(.12)	< .001		1.07	(.12)	< .001
σ^2^ between-person (BP)	.54	(.08)	< .001		.54	(.08)	< .001	.47	(.08)	< .001		.47	(.08)	< .001
σ^2^ passive use (BP)					.02	(.05)	.335					.06	(.04)	.068
σ^2^ active use (BP)					.05	(.07)	.217					.04	(.04)	.142
**Model fit**														
Deviance	6,503.54				6,501.67			3,287.59				3,279.66		
AIC	6,515.54				6,517.67			3,299.59				3,295.33		
BIC	6,549.48				6,562.91			3,329.47				3,335.17		
Chi^2^ (df)					1.37 (2)		.503					19.92 (2)		< .001

For investigating the categorical associations, the passive and active use predictors were dummy coded (passive use: 0 = no passive use of Instagram; 1 = passive use of Instagram; and active use: 0 = no active use of Instagram; 1 = active use of Instagram, respectively). WP = within-person; BP = between-person. All predictors were person-mean centered. Models for the duration of use only include assessments during which participants had used Instagram, either actively or passively. *p*-values of the fixed part of the model are two-sided, *p*-values of the random part of the model are one-sided.

**Table 4:** Within-person associations between adolescents’ use of WhatsApp and well-being.

	Categorical associations Use versus no use (*N* participants = 63; *N* assessments = 2,154)							Dose–response associations Duration of use (*N* participants = 62; *N* assessments = 1,179)						
	Model 5A Fixed effects				Model 5B Random effects			Model 6A Fixed effects				Model 6B Random effects		
	*B*	(*SE*)	*p*	β	*B*	(*SE*)	*p*	*B*	(*SE*)	*p*	β	*B*	(*SE*)	*p*
**Fixed part**														
Intercept	5.60	(.10)	< .001	7.62	5.60	(.11)	< .001	5.69	(.09)	< .001	7.62	5.69	(.09)	< .001
Assessment (WP)	.08	(.04)	.023	.09	.09	(.04)	.019	.09	(.04)	.049	.09	.09	(.04)	.047
Passive use (WP)	.16	(.05)	.001	.14	.22	(.09)	.022	.18	(.05)	< .001	.14	.18	(.04)	< .001
Active use (WP)	.06	(.07)	.364	.06	.02	(.09)	.810	− .05	(.06)	.444	.06	− .05	(.05)	.258
**Random part**														
σ^2^ residual (WP)	1.16	(.11)	< .001		1.15	(.11)	< .001	1.10	(.11)	< .001		1.10	(.12)	< .001
σ^2^ between-person (BP)	.54	(.08)	< .001		.54	(.08)	< .001	.47	(.09)	< .001		.47	(.09)	< .001
σ^2^ passive use (BP)					.04	(.08)	.296					< .01	(.01)	.440
σ^2^ active use (BP)					.02	(.05)	.360					< .01	(.01)	.310
**Model fit**														
Deviance	6,615.24				6,613.47			3,590.86				3,591.40		
AIC	6,627.24				6,629.47			3,602.86				3,607.40		
BIC	6,661.29				6,674.87			3,633.30				3,647.98		
Chi^2^ (df)					1.14 (2)		.566					2.81 (2)		.245

For investigating the categorical associations, the passive and active use predictors were dummy coded (passive use: 0 = no passive use of WhatsApp; 1 = passive use of WhatsApp; and active use: 0 = no active use of WhatsApp; 1 = active use of WhatsApp, respectively). WP = within-person; BP = between-person. All predictors were person-mean centered. Models for the duration of use only include assessments during which participants had used WhatsApp, either actively or passively. *p*-values of the fixed part of the model are two-sided, *p*-values of the random part of the model are one-sided.

#### Overall social media use.

The model with the categorical predictors (see Table 2; Model 1A) showed that, on average, there was no association between overall use and well-being: Adolescents’ well-being did not increase or decrease at moments when they had used social media, either in a passive or active way. However, evidence was found that the association of passive (but not active) social media use with well-being differed from adolescent to adolescent (Model 1B), with effect sizes ranging from − 0.24 to 0.68. For 44.26% of the adolescents the association was non-existent to small (− 0.10 < *r* < 0.10). However, for 45.90% of the adolescents there was a weak (0.10 < *r* < 0.20; 8.20%), moderate (0.20 < *r* < 0.30; 22.95%) or even strong positive (*r* ≥ 0.30; 14.75%) association between overall passive social media use and well-being, and for almost one in ten (9.84%) adolescents there was a weak (− 0.20 < *r* < − 0.10; 6.56%) or moderate negative (− 0.30 < *r* < − 0.20; 3.28%) association.

The model with continuous predictors (Model 2A) showed that, on average, there was a significant dose–response association for active use. At moments when adolescents had used social media, the time they spent *actively* (but not passively) using social media was positively associated with well-being: Adolescents felt better at moments when they had spent more time sending messages, posting, or sharing something on social media. The associations of the time spent actively and passively using social media with well-being did not differ across adolescents (Model 2B).

#### Instagram use

As shown in Model 3A in Table 3, on average, there was a significant categorical association between *passive* (but not active) Instagram use and well-being: Adolescents experienced an increase in well-being at moments when they had *passively* used Instagram (i.e., viewing posts/stories of others). Adolescents did not experience an increase or decrease in well-being when they had *actively* used Instagram. The associations of passive and active Instagram use with well-being did not differ across adolescents (Model 3B).

**Figure 1:**
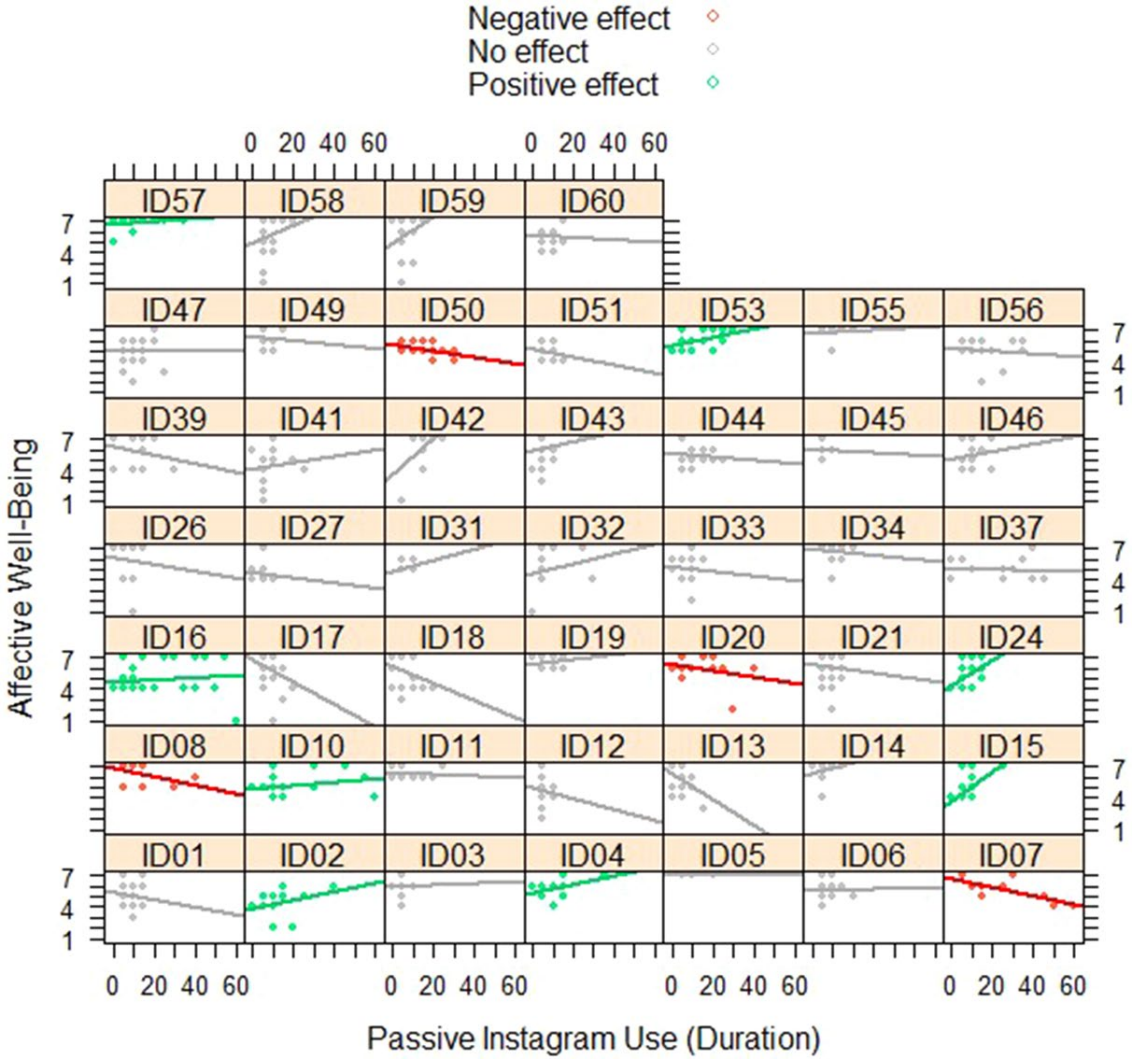
The dose–response association between passive Instagram use (in minutes per hour) and affective well-being for each individual adolescent (n = 46). Red lines represent significant negative within-person associations, green lines represent significant positive within-person associations, and gray lines represent non-significant within-person associations. A graph was created for each participant who had completed at least 10 assessments. A total of 13 participants were excluded because they had completed less than 10 assessments of passive Instagram use. In addition, one participant was excluded because no graph could be computed, since this participant's passive Instagram use was constant across assessments.

On average, no significant dose–response association was found for Instagram use (Model 4A): At moments when adolescents had used Instagram, the time adolescents spent using Instagram (either actively or passively) was not associated with their well-being. However, evidence was found that the association of the time spent *passively* using Instagram differed from adolescent to adolescent (Model 4B), with effect sizes ranging from − 0.48 to 0.27. For most adolescents (73.91%) the association was non-existent to small (− 0.10 < *r* < 0.10), but for almost one in five adolescents (17.39%) there was a weak (0.10 < *r* < 0.20; 10.87%) or moderate (0.20 < *r* < 0.30; 6.52%) positive association, and for almost one in ten adolescents (8.70%) there was a weak (− 0.20 < *r* < − 0.10; 2.17%), moderate (− 0.30 < *r* < − 0.20; 4.35%), or strong (*r* ≤ − 0.30; 2.17%) negative association. Figure 1 illustrates these differences in the dose–response associations.

#### WhatsApp use

As shown in Model 5A in Table 4, just as for Instagram, we found that, on average, there was a significant categorical association between *passive* (but not active) WhatsApp use and well-being: Adolescents reported that they felt better at moments when they had *passively* used WhatsApp (i.e., read WhatsApp messages). For *active* WhatsApp use, no significant association was found. Also, in line with the results for Instagram use, no differences were found regarding the associations of active and passive WhatsApp use (Model 5B).

In addition, a significant dose–response association was found for *passive* (but not active) use (Model 6A). At moments when adolescents had used WhatsApp, we found that, on average, the time adolescents spent *passively* using WhatsApp was positively associated with well-being: Adolescents felt better at moments when they had spent more time reading WhatsApp messages. The time spent *actively* using WhatsApp was not associated with well-being. No differences were found in the dose–response associations of active and passive WhatsApp use (Model 6B).

## Discussion

This preregistered study investigated adolescents’ unique susceptibility to the effects of social media. We found that the associations of passive (but not active) social media use with well-being differed substantially from adolescent to adolescent, with effect sizes ranging from moderately negative (− 0.24) to strongly positive (0.68). While 44.26% of adolescents did not feel better or worse if they had passively used social media, 45.90% felt better, and a small group felt worse (9.84%). In addition, for Instagram the majority of adolescents (73.91%) did not feel better or worse when they had spent more time viewing post or stories of others, whereas some felt better (17.39%), and others (8.70%) felt worse.

These findings have important implications for social media effects research, and media effects research more generally. For decades, researchers have argued that people differ in their susceptibility to the effects of media^17^, leading to numerous investigations of such differential susceptibility. These investigations have typically focused on moderators, based on variables such as sex, age, or personality. Yet, over the years, studies have shown that such moderators appear to have little power to explain how individuals differ in their susceptibility to media effects, probably because a group-differential approach does not account for the possibility that media users may differ across a range of factors, that are not captured by only one (or a few) investigated moderator variables.

By providing insights into each individual’s unique susceptibility, the findings of this study provide an explanation as to why, up until now, most media effects research has only found small effects. We found that the majority of adolescents do not experience any short-term changes in well-being related to their social media use. And if they do experience any changes, these are more often positive than negative. Because only small subsets of adolescents experience small to moderate changes in well-being, the true effects of social media reported in previous studies have probably been diluted across heterogeneous samples of individuals that differ in their susceptibility to media effects (also see^30^). Several scholars have noted that overall effect sizes may mask more subtle individual differences^14, 15^, which may explain why previous studies have typically reported small or no effects of social media on well-being or indicators of well-being^6, 11–13^. The current study seems to confirm this assumption, by showing that while the overall effect sizes are small at best, the person-specific effect sizes vary considerably, from tiny and small to moderate and strong.

As called upon by other scholars^5, 31^, we disentangled the associations of active and passive use of social media. Research among young adults found that passive (but not active) social media use is associated with lower levels of affective well-being^29^. In line with these findings, the current study shows that active and passive use yielded different associations with adolescents’ affective well-being. Interestingly though, in contrast to previous findings among adults, our study showed that, on average, passive use of Instagram and WhatsApp seemed to enhance rather than decrease adolescents’ well-being. This discrepancy in findings may be attributed to the fact that different mechanisms might be involved. Verduyn and colleagues^29^ found that passive use of Facebook undermines adults’ well-being by enhancing envy, which may also explain the decreases in well-being found in our study among a small group of adolescents. Yet, adolescents who felt better by passively using Instagram and WhatsApp, might have felt so because they experienced enjoyment. After all, adolescents often seek positive content on social media, such as humorous posts or memes^32^. Also, research has shown that adolescents mainly receive positive feedback on social media^33^. Hence, their passive Instagram and WhatsApp use may involve the reading of positive feedback, which may explain the increases in well-being.

Overall, the time spent passively using WhatsApp improved adolescents’ well-being. This did not differ from adolescent to adolescent. However, the associations of the time spent passively using Instagram with well-being did differ from adolescent to adolescent. This discrepancy suggests that not all social media uses yield person-specific effects on well-being. A possible explanation may be that adolescents’ responses to WhatsApp are more homogenous than those to Instagram. WhatsApp is a more private platform, which is mostly used for one-to-one communication with friends and acquaintances^26^. Instagram, in contrast, is a more public platform, which allows its users to follow a diverse set of people, ranging from best friends to singers, actors, and influencers^28^, and to engage in intimate communication as well as self-presentation and social comparison. Such diverse uses could lead to more varied, or even opposing responses, such as envy versus inspiration.

### Limitations and directions for future research

The current study extends our understanding of differential susceptibility to media effects, by revealing that the effect of social media use on well-being differs from adolescent to adolescent. The findings confirm our assumption that among the great majority of adolescents, social media use is unrelated to well-being, but that among a small subset, social media use is either related to decreases or increases in well-being. It must be noted, however, that participants in this study felt relatively happy, overall. Studies with more vulnerable samples, consisting of clinical samples or youth with lower social-emotional well-being may elicit different patterns of effects^27^. Also, the current study focused on affective well-being, operationalized as happiness. It is plausible that social media use relates differently with other types of well-being, such as cognitive well-being. An important next step is to identify *which* adolescents are particularly susceptible to experience declines in well-being. It is conceivable, for instance, that the few adolescents who feel worse when they use social media are the ones who receive negative feedback on social media^33^.

In addition, future ESM studies into the effects of social media should attempt to include one or more follow-up measures to improve our knowledge of the longer-term influence of social media use on affective well-being. While a week-long ESM is very common and applied in most earlier ESM studies^34^, a week is only a snapshot of adolescent development. Research is needed that investigates whether the associations of social media use with adolescents’ momentary affective well-being may cumulate into long-lasting consequences. Such investigations could help clarify whether adolescents who feel bad in the short term would experience more negative consequences in the long term, and whether adolescents who feel better would be more resistant to developing long-term negative consequences. And while most adolescents do not seem to experience any short-term increases or decreases in well-being, more research is needed to investigate whether these adolescents may experience a longer-term impact of social media.

While the use of different platforms may be differently associated with well-being, different types of use may also yield different effects. Although the current study distinguished between active and passive use of social media, future research should further differentiate between different activities. For instance, because passive use entails many different activities, from reading private messages (e.g., WhatsApp messages, direct messages on Instagram) to browsing a public feed (e.g., scrolling through posts on Instagram), research is needed that explores the unique effects of passive public use and passive private use. Research that seeks to explore the nuances in adolescents’ susceptibility as well as the nuances in their social media use may truly improve our understanding of the effects of social media use.

## Methods

### Participants

Participants were recruited via a secondary school in the south of the Netherlands. Our preregistered sampling plan set a target sample size of 100 adolescents. We invited adolescents from six classrooms to participate in the study. The final sample consisted of 63 adolescents (i.e., 42% consent rate, which is comparable to other ESM studies among adolescents; see, for instance^35, 36^). Informed consent was obtained from all participants and their parents. On average, participants were 15 years old (*M* = 15.12 years, *SD* = 0.51) and 54% were girls. All participants self-identified as Dutch, and 41.3% were enrolled in the prevocational secondary education track, 25.4% in the intermediate general secondary education track, and 33.3% in the academic preparatory education track.

### Procedure

The study was approved by the Ethics Review Board of the Faculty of Social and Behavioral Sciences at the University of Amsterdam and was performed in accordance with the guidelines formulated by the Ethics Review Board. The study consisted of two phases: A baseline survey and a personalized week-long experience sampling (ESM) study. In phase 1, researchers visited the school during school hours. Researchers informed the participants of the objective and procedure of the study and assured them that their responses would be treated confidentially. Participants were asked to sign the consent form. Next, participants completed a 15-min baseline survey. The baseline survey included questions about demographics and assessed which social media each adolescent used most frequently, allowing to personalize the social media questions presented during the ESM study in phase 2. After completing the baseline survey, participants were provided detailed instructions about phase 2.

In phase 2, which took place two and a half weeks after the baseline survey, a 7-day ESM study was conducted, following the guidelines for ESM studies provided by van Roekel and colleagues^34^. Aiming for at least 30 assessments per participant and based on an average compliance rate of 70 to 80% reported in earlier ESM studies among adolescents^34^, we asked each participant to complete a total of 42 ESM surveys (i.e., six 2-min surveys per day). Participants completed the surveys using their own mobile phone, on which the ESM software application Ethica Data was installed during the instruction session with the researchers (phase 1). Each 2-min survey consisted of 22 questions, which assessed adolescents’ well-being and social media use. Two open-ended questions were added to the final survey of the day, which asked about adolescents’ most pleasant and most unpleasant events of the day.

The ESM sampling scheme was semi-random, to allow for randomization and avoid structural patterns in well-being, while taking into account that adolescents were not allowed to use their phone during school time. The Ethica Data app was programmed to generate six beep notifications per day at random time points within a fixed time interval that was tailored to the school’s schedule: before school time (1 beep), during school breaks (2 beeps), and after school time (3 beeps). During the weekend, the beeps were generated during the morning (1 beep), afternoon (3 beeps), and evening (2 beeps). To maximize compliance, a 30-min time window was provided to complete each survey. This time window was extended to one hour for the first survey (morning) and two hours for the final survey (evening) to account for travel time to school and time spent on evening activities. The average compliance rate was 83.2%. A total of 2,155 ESM assessments were collected: Participants completed an average of 34.83 surveys (*SD* = 4.91) on a total of 42 surveys, which is high compared to previous ESM studies among adolescents^34^.

The questions of the ESM study were personalized based on the responses to the baseline survey. During the ESM study, each participant reported on his/her use of three different social media platforms: WhatsApp and either Instagram, Snapchat, YouTube, and/or the chat function of games (i.e., the most popular social media platforms among adolescents^28^). Questions about Instagram and WhatsApp use were only included if the participant had indicated in the baseline survey that s/he used these platforms at least once a week. If a participant had indicated that s/he used Instagram or WhatsApp (or both) less than once a week, s/he was asked to report on the use of Snapchat, YouTube, or the chat function of games, depending on what platform s/he used at least once a week. In addition to Instagram and WhatsApp, questions were asked about a third platform, that was selected based on how frequently the participant used Snapchat, YouTube, or the chat function of games (i.e., at least once a week). This resulted in five different combinations of three platforms: Instagram, WhatsApp, and Snapchat (47 participants); Instagram, WhatsApp, and YouTube (11 participants); Instagram, WhatsApp, and chatting via games (2 participants); WhatsApp, Snapchat, and YouTube (1 participant); and WhatsApp, YouTube, and chatting via games (2 participants).

### Frequency of social media use

In the baseline survey, participants were asked to indicate how often they used and checked Instagram, WhatsApp, Snapchat, YouTube, and the chat function of games, using response options ranging from 1 (*never*) to 7 (*more than 12 times per day*). These platforms are the five most popular platforms among Dutch 14- and 15-year-olds^28^. Participants’ responses were used to select the three social media platforms that were assessed in the personalized ESM study.

### Duration of social media use

In the ESM study, duration of active and passive social media use was measured by asking participants how much time in the past hour they had spent actively and passively using each of the three platforms that were included in the personalized ESM surveys. Response options ranged from *0* to *60 min*, with 5-min intervals. To measure active Instagram use, participants indicated how much time in the past hour they had spent (a) “posting on your feed or sharing something in your story on Instagram” and (b) “sending direct messages/chatting on Instagram.” These two items were summed to create the variable duration of active Instagram use. Sum scores exceeding 60 min (only 0.52% of all assessments) were recoded to 60 min. To measure duration of passive Instagram use, participants indicated how much time in the past hour they had spent “viewing posts/stories of others on Instagram.” To measure the use of WhatsApp, Snapchat, YouTube and game-based chatting, we asked participants how much time they had spent “sending WhatsApp messages” (active use) and “reading WhatsApp messages” (passive use); “sending snaps/messages or sharing something in your story on Snapchat” (active use) and “viewing snaps/stories/messages from others on Snapchat” (passive use); “posting YouTube clips” (active use) and “watching YouTube clips” (passive use); “sending messages via the chat function of a game/games” (active use) and “reading messages via the chat function of a game/games” (passive use). Duration of active and passive overall social media use were created by summing the responses across the three social media platforms for active and passive use, respectively. Sum scores exceeding 60 min (2.13% of all assessments for active overall use; 2.90% for passive overall use) were recoded to 60 min. The duration variables were used to investigate whether the time spent actively or passively using social media was associated with well-being (dose–response associations).

### Use/no use of social media

Based on the duration variables, we created six dummy variables, one for active and one for passive overall social media use, one for active and one for passive Instagram use, and one for active and one for passive WhatsApp use (0 = *no active use* and 1 = *active use*, and 0 = *no passive use* and 1 = *passive use*, respectively). These dummy variables were used to investigate whether the use of social media, irrespective of the duration of use, was associated with well-being (categorical associations).

### Well-being

Consistent with previous ESM studies^19, 20^, we measured affective well-being using one item, asking “How happy do you feel right now?” at each assessment. Adolescents indicated their response to the question using a 7-point scale ranging from 1 (*not at all*) to 7 (*completely*), with 4 (*a little*) as the midpoint. Convergent validity of this item was established in a separate pilot ESM study among 30 adolescents conducted by the research team of the fourth author: The affective well-being item was strongly correlated with the presence of positive affect and absence of negative affect (assessed by a 10-item positive and negative affect schedule for children; PANAS-C) at both the between-person (positive affect: *r* = 0.88, p < 0.001; negative affect: *r* = − 0.62, p < 0.001) and within-person level (positive affect: *r* = 0.74, p < 0.001; negative affect:* r* = − 0.58, p < 0.001).

### Statistical analyses

Before conducting the analyses, several validation checks were performed (see^34^). First, we aimed to only include participants in the analyses who had completed more than 33% of all ESM assessments (i.e., at least 14 assessments). Next, we screened participants’ responses to the open questions for unserious responses (e.g., gross comments, jokes). And finally, we inspected time series plots for patterns in answering tendencies. Since all participants completed more than 33% of all ESM assessments, and no inappropriate responses or low-quality data patterns were detected, all participants were included in the analyses.

Following our preregistered analysis plan, we tested the proposed associations in a series of multilevel models. Before doing so, we tested the homoscedasticity and linearity assumptions for multilevel analyses^37^. Inspection of standardized residual plots indicated that the data met these assumptions (plots are available on OSF at https://osf.io/nhks2). We specified separate models for overall social media use, use of Instagram, and use of WhatsApp. To investigate to what extent adolescents’ well-being would vary depending on whether they had actively or passively used social media/Instagram/WhatsApp or not during the past hour (categorical associations), we tested models including the dummy variables as predictors (active use versus no active use, and passive use versus no passive use; models 1, 3, and 5). To investigate whether, at moments when adolescents had used social media/Instagram/WhatsApp during the past hour, their well-being would vary depending on the duration of social media/Instagram/WhatsApp use (dose–response associations), we tested models including the duration variables as predictors (duration of active use and duration of passive use; models 2, 4, and 6). In order to avoid negative skew in the duration variables, we only included assessments during which adolescents had used social media in the past hour (overall, Instagram, or WhatsApp, respectively), either actively or passively. All models included well-being as outcome variable. Since multilevel analyses allow to include all available data for each individual, no missing data were imputed and no data points were excluded.

We used a model building approach that involved three steps. In the first step, we estimated an intercept-only model to assess the relative amount of between- and within-person variance in affective well-being. We estimated a three-level model in which repeated momentary assessments (level 1) were nested within adolescents (level 2), who, in turn, were nested within classrooms (level 3). However, because the between-classroom variance in affective well-being was small (i.e., 0.4% of the variance was explained by differences between classes), we proceeded with estimating two-level (instead of three-level) models, with repeated momentary assessments (level 1) nested within adolescents (level 2).

In the second step, we assessed the within-person associations of well-being with (a) overall active and passive social media use (i.e., the total of the three platforms), (b) active and passive use of Instagram, and (c) active and passive use of WhatsApp, by adding fixed effects to the model (Models 1A-6A). To facilitate the interpretation of the associations and control for the effects of time, a covariate was added that controlled for the *n*th assessment of the study week (instead of the *n*th assessment of the day, as preregistered). This so-called detrending is helpful to interpret within-person associations as correlated fluctuations beyond other changes in social media use and well-being^38^. In order to obtain within-person estimates, we person-mean centered all predictors^38^. Significance of the fixed effects was determined using the Wald test.

In the third and final step, we assessed heterogeneity in the within-person associations by adding random slopes to the models (Models 1B-6B). Significance of the random slopes was determined by comparing the fit of the fixed effects model with the fit of the random effects model, by performing the Satorra-Bentler scaled chi-square test^39^ and by comparing the Bayesian information criterion (BIC^40^) and Akaike information criterion (AIC^41^) of the models. When the random effects model had a significantly better fit than the fixed effects model (i.e., pointing at significant heterogeneity), variance components were inspected to investigate whether heterogeneity existed in the association of either active or passive use. Next, when evidence was found for significant heterogeneity, we computed person-specific effect sizes, based on the random effect models, to investigate what percentages of adolescents experienced better well-being, worse well-being, and no changes in well-being. In line with Keijsers and colleagues^42^ we only included participants who had completed at least 10 assessments. In addition, for the dose–response associations, we constructed graphical representations of the person-specific slopes, based on the person-specific effect sizes, using the *xyplot* function from the lattice package in R^43^.

Three improvements were made to our original preregistered plan. First, rather than estimating the models with multilevel modelling in R^43^, we ran the preregistered models in Mplus^44^. Mplus provides standardized estimates for the fixed effects models, which offers insight into the effect sizes. This allowed us to compare the relative strength of the associations of passive versus active use with well-being. Second, instead of using the maximum likelihood estimator, we used the maximum likelihood estimator with robust standard errors (MLR), which are robust to non-normality. Sensitivity tests, uploaded on OSF (https://osf.io/nhks2), indicated that the results were almost identical across the two software packages and estimation approaches. Third, to improve the interpretation of the results and make the scales of the duration measures of social media use and well-being more comparable, we transformed the social media duration scores (0 to 60 min) into scales running from 0 to 6, so that an increase of 1 unit reflects 10 min of social media use. The model estimates were unaffected by this transformation.

### Reporting summary

Further information on the research design is available in the Nature Research Reporting Summary linked to this article.

## Data Availability

The dataset generated and analysed during the current study is available in Figshare^45^. The preregistration of the design, sampling and analysis plan, and the analysis scripts used to analyse the data for this paper are available online on the Open Science Framework website (https://osf.io/nhks2).
